# Correspondence between symptom development of *Colletotrichum graminicola* and fungal biomass, quantified by a newly developed qPCR assay, depends on the maize variety

**DOI:** 10.1186/s12866-016-0709-4

**Published:** 2016-05-23

**Authors:** Fabian Weihmann, Iris Eisermann, Rayko Becher, Jorrit-Jan Krijger, Konstantin Hübner, Holger B. Deising, Stefan G. R. Wirsel

**Affiliations:** Institut für Agrar- und Ernährungswissenschaften, Naturwissenschaftliche Fakultät III, Martin-Luther-Universität Halle-Wittenberg, Betty-Heimann-Str. 3, D-06120 Halle (Saale), Germany; Interdisziplinäres Zentrum für Nutzpflanzenforschung (IZN), Martin-Luther-Universität Halle-Wittenberg, Betty-Heimann-Str. 3, D-06120 Halle (Saale), Germany

**Keywords:** *Colletotrichum graminicola*, *Zea mays*, Maize, Virulence, PTI, ETI, Papilla

## Abstract

**Background:**

Penetration attempts of the hemibiotroph *Colletotrichum graminicola* may activate PAMP-triggered immunity (PTI) on different cultivars of *Zea mays* to different extent. However, in most events, this does not prevent the establishment of a compatible pathogenic interaction. In this study, we investigate the extent to which the host variety influences PTI. Furthermore, we assess whether visual disease symptoms occurring on different maize varieties reliably reflect fungal biomass development *in planta* as determined by qPCR and GFP tracing.

**Results:**

Employing a set of four maize varieties, which were selected from a panel of 27 varieties, for in-depth assessment of pathogenesis of the wild type strain of *C. graminicola,* revealed considerable differences in susceptibility as evidenced by symptom severity that decreased from variety Golden Jubilee to Mikado to Farmtop to B73. However, a newly developed qPCR assay and microscopical observation of a GFP-labelled strain showed that disease symptoms are in some instances inconsistent when compared with other indicators of susceptibility. Of the four varieties assessed, either Golden Jubilee, Mikado and B73, or Golden Jubilee, Farmtop and B73 showed a direct correlation between symptom and fungal biomass development. In a pairwise comparison, however, Mikado and Farmtop showed an inverse correlation for these features.

**Conclusions:**

The genotype of maize contributes to the severity of symptoms resulting from an infection with *C. graminicola*. Partially, this may be attributed to the extent of PTI activated in different varieties, as reflected by papilla formation. Furthermore, when evaluating the susceptibility of a variety, it should be considered that symptom severity must not have to reflect the extent of fungal growth in the infected tissue.

**Electronic supplementary material:**

The online version of this article (doi:10.1186/s12866-016-0709-4) contains supplementary material, which is available to authorized users.

## Background

The genus *Colletotrichum* comprises many important pathogenic fungi that typically cause anthracnose disease symptoms on aerial plant surfaces or post-harvest rots [1, 2]. Many species of that genus show a hemibiotrophic lifestyle that is characterized by a short initial biotrophic phase and a subsequent switch to necrotrophic proliferation. The causal agent of anthracnose leaf blight and stalk rot of maize, *Colletotrichum graminicola* (Ces.) G. W. Wils. (teleomorph *Glomerella graminicola* Politis), can potentially cause considerable losses of up to one billion U.S. dollars in the Americas per year [3]. Investigation of this pathosystem has been advanced by the recent annotations of the genomes of both the host and the fungus [4, 5].

Several plant defence responses have been characterized for the *C. graminicola* - maize pathosystem. Even in the compatible interaction, the invading pathogen does not remain fully undetected since host reactions such as the formation of papillae (callose-containing cell wall appositions), phytoalexins and H_2_O_2_, do occur at some degree during the biotrophic phase [6–9]. These observations led to the suggestion that the pathogen escapes such defence reactions by switching to necrotrophy [7]. This may imply that in contrast to obligate biotrophs the secreted effectors of this hemibiotroph may not allow for a sufficiently strong and lasting suppression of the host´s defence systems. Nevertheless, it is assumed that the establishment of a biotrophic phase may provide some advantages to the fungus. It is thus crucial to investigate the roles of effector proteins of *C. graminicola* during the different phases of pathogenesis.

However, assessment of mutants in candidate genes can be difficult. Besides the general problem of consistently quantifying symptom development, another challenge comes from the observation made in other pathosystems that disease severity may not always correlate with fungal biomass development within infected host tissues. This is for instance important in a scenario where a fungus proliferates *in planta* without causing apparent symptoms as in endophytes. On the other hand, the genotype of the host may also affect fungal virulence and the extent of symptom development.

Work comparing stalk rot in a susceptible and a resistant maize hybrid uncovered a correlation between the extent of macroscopic symptoms and ergosterol levels, as an indicator of fungal biomass [10]. However, an earlier study suggested that ergosterol contents often do not correlate well with fungal biomass [11]. Thus, an additional, convenient method such as qPCR would be helpful to assess reliably fungal biomass development in maize anthracnose.

We used the ITS2 region, which is a part of the fungal rDNA cluster, for the development and validation of a qPCR assay that is not only very sensitive but also highly specific so that interference with host DNA does not occur. This qPCR assay was employed to determine whether macroscopic symptom development of *C. graminicola* on four maize varieties, which were chosen from 27 varieties to represent gradually increasing levels of susceptibility, is reflected by fungal biomass accumulation. In addition, we employed a GFP-expressing *C. graminicola* strain to examine fungal proliferation *in planta* and compared this to the qPCR results. These experiments confirmed that fungal proliferation in the tested maize varieties diverges especially during early pathogenesis.

## Results

### Development of a qPCR assay to quantify *C. graminicola* biomass *in planta*

To employ proper quantification of fungal gDNA as a measure of fungal biomass in infected plant tissue, we initially developed and evaluated an optimised qPCR assay. This has been achieved in four subsequent steps as follows.

**Fig. 1:**
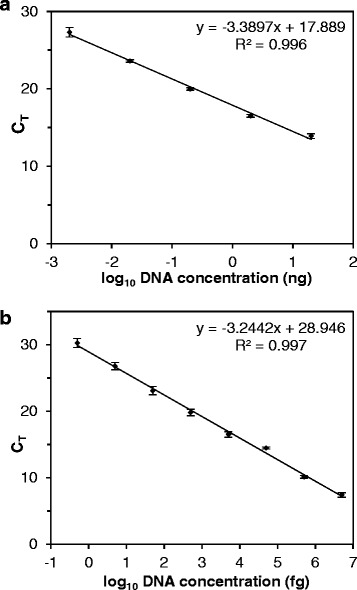
Calibration curves of qPCR. Data provide means, error bars standard deviations. A) gDNA of *C. graminicola* isolated from three independent cultures was serially diluted twice in a constant amount of 10 ng of gDNA of *Z. mays* Mikado. Each dilution was measured twice, giving a total of 12 values for each concentration. B) Plasmid pUC18 was serially diluted in 10 ng of gDNA of *Z. mays* and measured thrice, giving three values for each concentration

**Fig. 2:**
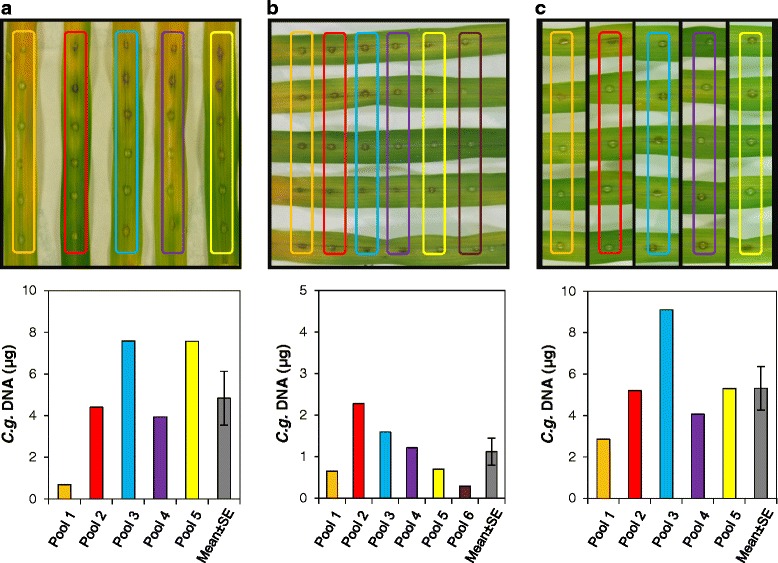
Effects of tissue pooling on qPCR results. Upper part shows infection sites on detached maize leaves at 96 hpi and the indicated pooling schemes for DNA extraction. The lower part shows the results of the corresponding qPCRs that used 10 ng of total DNA from each pool. An individual column represents the absolute amount of fungal DNA determined in the leaf disc pool that is boxed with the same colour in the upper part. Grey columns represent the calculated means and standard errors (SE) for data merged from all pools. **a** Six excised tissue samples were combined from individual leaves. **b** Excised tissue samples were combined from five different leaves each carrying six infection sites, according to the position of the inoculum on the leaf. **c** Excised tissue samples were combined from five leaves each carrying a single infection site in its middle

**Fig. 3:**
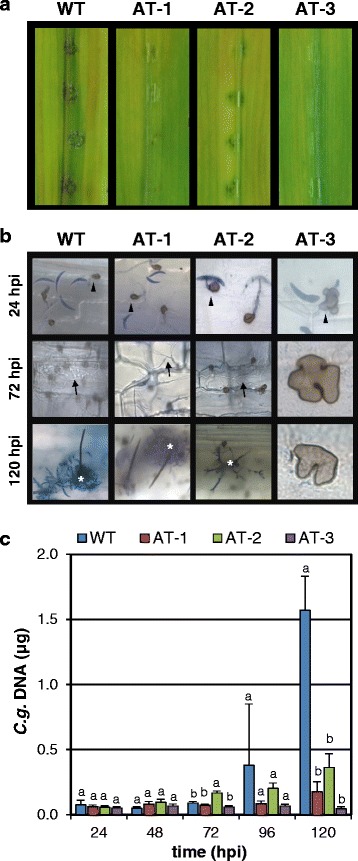
Effects of mutations affecting virulence on qPCR results. Three ATMT mutants (AT-1, AT-2 and AT-3) were inoculated on maize variety Mikado and compared to the wild type reference. **a** Symptoms occurring at 120 hpi. **b** Light microscopy of infection sites after staining with aniline blue. Arrowheads indicate appressoria, arrows invasive secondary hyphae and asterisks acervuli. Photographs shown in **a** and **b** provide representative results from five repeated experiments each using four leaves per variant. **c** Result of qPCR using 10 ng of total DNA as template. Columns represent means of five independent experiments. For each experiment, eight leaf discs excised from individual leaves carrying a single inoculation site at their middle were pooled. Error bars indicate standard errors. Letters assess variation between the strains at a given time, which was determined by multiple comparisons using the Student-Newman-Keuls test. Different letters indicate significant differences at *P* < 0.05

(i) *Primer design and evaluation.* Primer Cg_ITS2-F1.1 and Cg_ITS2-R1 target the ITS2 region of the rDNA cluster with high specificity and provide high sensitivity, due to the presence of a predicted number of 60 rDNA repeats (http://www.broadinstitute.org/annotation/genome/colletotrichum_group/GenomeStats.html), for the quantification of gDNA of *C. graminicola*. PCR using these primers amplified a 98 bp fragment from gDNA of *C. graminicola*, but showed no products with the gDNA of *Z. mays* and pUC18 (Additional file 1A). In the following experiments, the plasmid served as a spike-in control where a known quantity of DNA was added to maize tissue samples just before DNA extraction. This has been used to compare the experimentally determined quantity of pUC18 to derive sample-specific correction factors that allowed proportionally correcting the experimentally determined quantity of fungal DNA for preparation-based inaccuracies. Sequencing of the PCR fragment confirmed the identity of the targeted ITS2 region (not shown). In addition, the primer pair M13new-For and M13new-Rev amplified a fragment of 105 bp from pUC18 DNA, but did not do so with DNA of the fungus or its host (Additional file 1B).
(ii) *Assessing calibration curves.* Subsequent to validation of primer specificity, for both primer pairs qPCR calibration curves have been established with serial dilutions of fungal gDNAs made in 10 ng of *Z. mays* gDNA (containing ca. 4.3 x 10^3^ copies) to simulate different degrees of colonisation of maize leaves by *C. graminicola*. We found a linear dynamic range in the ITS2 assay from 2 pg to 20 ng of *C. graminicola* gDNA, corresponding to ca. 2.2 x 10^2^ to ca. 2.2 x 10^6^ ITS2 copies (assuming 60 rDNA repeats per genome) in the maize DNA background (Fig. 1a). The PCR efficiency was 97 %. Calibration curve analysis of the assay for pUC18 resulted in a linear dynamic range from 0.5 fg to 5 ng of template DNA (ca. 1.8 x 10^2^ to ca. 1.8 x 10^9^ copies; Fig. 1b); the PCR efficiency was 103 %.
(iii) *Tissue sampling optimisation.* Compared to spray inoculation of whole plants, point-inoculation of a defined number of spores onto detached leaves allows better control of variation of the resulting macroscopic symptoms in biological replicates. However, some variability remains and, thus, might limit the power of qPCR analyses. Therefore, using the qPCR assay described above we quantified variations of fungal development between different leaves and between different positions of inoculation on an individual leaf. To evaluate these variations, detached maize leaves were inoculated with six droplets set equidistantly along the segments, incubated for 96 h and analysed in two ways. Infection sites were excised and then either pooled per leaf (Fig. 2a) or pooled across different leaves according to the position of the inoculum (Fig. 2b). For both pooling strategies, qPCR-based calculation of fungal gDNA contents resulted in some variation. Whereas pools representing individual leaves exhibited random variation (Fig. 2a), pools representing distinct regions of infected leaves (Fig. 2b) showed a more systematic variation since tissue samples from the margins contained less fungal DNA than those from the middle. Moreover, in the across-leaves comparison, extreme values varied only by a factor of about three, whereas in the within-leaves comparison, extreme values varied by a factor of about ten. Variation coefficients in these experiments were 0.65 and 0.6, respectively. To reduce variations we tested another sampling scheme where only the middle of each leaf carried a single inoculation point. When using pools for qPCR analysis that comprised five individual infection sites on different leaves, the variation coefficient decreased to 0.44 (Fig. 2c). Thus, this optimised sampling strategy was used in all subsequent experiments.
(iv) *Evaluation of the new qPCR assay.* To test the resolving power of the optimised qPCR assay, we compared the *in planta* biomass accumulation of the *C. graminicola* wild type strain with three previously recovered *Agrobacterium tumefaciens*-mediated transformation (ATMT) mutants [12] that were further characterised here. Southern blot analyses confirmed that each of the three mutants possessed a single T-DNA integration (not shown). The mutant strains, designated AT-1, AT-2 and AT-3, were chosen because they exhibited gradual reductions of virulence, i.e. visually assessable anthracnose symptoms on infected maize leaves (Fig. 3a). The infection process of these mutants was examined microscopically (Fig. 3b). At 24 hpi, conidia of all strains had germinated and differentiated appressoria. However, at 24 hpi strain AT-3 had formed only 30 % of the appressoria formed by the wild type strain, a difference that was statistically significant (Student's *t*-test, *p* ≤ 0.05). Most of the few appressoria of this strain appeared deformed (Fig. 3b). The development of strain AT-3 appeared arrested at the appressorial stage suggesting a failure to penetrate the plant epidermis. In contrast, strains AT-1 and AT-2 progressed through morphogenesis similar to the wild type. They showed secondary necrotrophic hyphae, conidiophores and setae at 72 hpi and 120 hpi, respectively (Fig. 3b).

Results from qPCR corresponded with the macroscopic and microscopic observations (Fig. 3c). The amount of DNA of strain AT-3 did not increase significantly over time, supporting the idea that this mutant had a penetration arrest and that it was therefore unable to propagate *in planta*. In the wild type and in mutant strains AT-1 and AT-2, fungal DNA levels developed uniformly from 24 to 72 hpi. From 72 hpi onwards, i.e. with the microscopically observed establishment of the necrotrophic infection stage, fungal proliferation within the maize leaves considerably accelerated for the wild type strain. In contrast, biomass of strains AT-1 and AT-2 increased much slower during this phase and that of AT-3 did not increase at all. At 120 hpi, the differences between the wild type and all mutants were statistically significant and amounted to factors of eight for AT-1, four for AT-2 and 34 for AT-3 (Fig. 3c). These experiments confirmed that the newly developed qPCR assay described here reliably quantifies fungal DNA contents in infected host tissues and is thus able to monitor fungal development altered by mutations affecting virulence.

### Anthracnose symptom development on different maize varieties

**Fig. 4:**
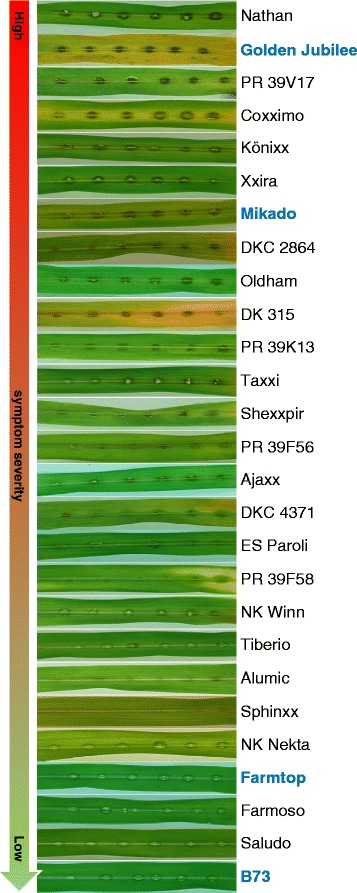
Screening of maize varieties for susceptibility to *C. graminicola.* Symptoms as occurring at 96 hpi. For each host variety, representative leaves are shown. Highlighted varieties were chosen for further experiments. This experiment was performed twice, each using three plants per variety. Leaves shown are representative results

To select a set of maize varieties with clear differences in disease susceptibility for subsequent analysis of a possible correlation between macroscopic symptoms and the underlying fungal biomass development, we evaluated 27 different maize varieties by detached leaf infection assays using the *C. graminicola* wild type strain (Additional file 2). The extent to which symptoms developed differed remarkably among the varieties tested (Fig. 4). At 4 dpi, 22 varieties showed black-coloured lesions at the inoculation sites, which harboured acervuli with melanised setae. However, among these varieties symptom intensity differed with regard to lesion size and level of discoloration. The remaining five maize varieties (NK Nekta, Farmoso, Farmtop, Saludo and B73) did not clearly show such macroscopic symptoms at 4 dpi.

**Fig. 5:**
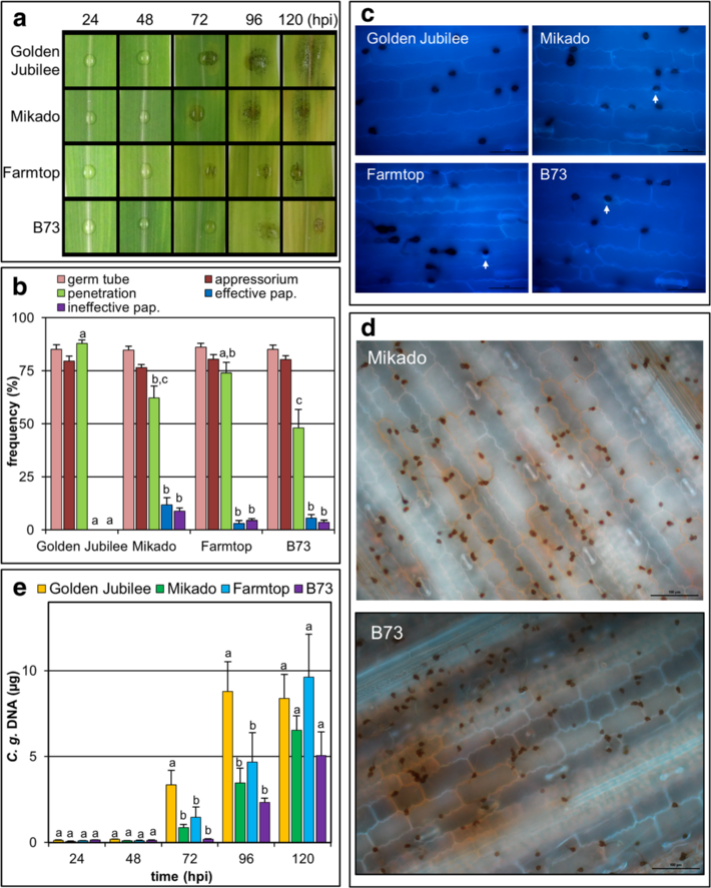
Influence of host genotype on symptoms, pathogenesis and qPCR results. Comparison of pathogenesis of the wild type reference strain on four maize varieties. **a**) Representative infection sites as observed at the indicated time points. **b**) Quantitation of fungal morphogenesis and host defence reactions. Columns give means of four biological replicates performed in different weeks, each in three technical replicates representing different leaves. On each leaf, infection structures developing from 100 conidia and the corresponding host responses were counted. Error bars provide standard errors. Letters assess variation between host varieties at 2 dpi, which was determined by multiple comparisons using the Student-Newman-Keuls test. Different letters indicate significant differences at *P* < 0.05. **c**) Fluorescence microscopy using UV2A filter exhibits whitish papillae and dark appressoria (600×). Arrows indicate papillae. Bars represent 50 μm. **d**) Fluorescence microscopy using UV2A filter and autowhite function on DAB-stained tissues exhibits sites of hydrogen peroxide production (200×). Results from the other two varieties were similar to those shown. Bars represent 100 μm. **e**) Results of qPCR using 10 ng of total DNA as template. Each column represents the absolute amount of fungal DNA that was averaged from five leaf disc pools. Each pool comprised eight leaf discs excised from individual leaves carrying a single inoculation site at their middle. Error bars indicate standard errors. Letters assess variation between host varieties at a given time as determined by the Student-Newman-Keuls test. Different letters indicate significant differences at *P* < 0.05

We chose four maize varieties strongly differing in disease susceptibility, i.e. Golden Jubilee, Mikado, Farmtop and B73 (highlighted in Fig. 4) to monitor the time-course of symptom development from 24 to 120 hpi (Fig. 5a). As seen above, the highly susceptible varieties Golden Jubilee and Mikado showed clear disease symptoms at 96 hpi. The former variety allowed for the fastest symptom development out of the four chosen. At 120 hpi, the anthracnose symptoms were more prominent on Golden Jubilee than on Mikado. At 120 hpi, weaker symptoms appeared also on Farmtop but almost none on B73 (Fig. 5a). In addition, we quantified microscopically fungal pathogenesis and defences of the four maize varieties. The ability to form germ tubes and appressoria on leaves was not much affected by the variety inoculated (Fig. 5b). However, host defence reactions differed between the varieties and this influenced the efficiency at which the fungus penetrated epidermal cells (Fig. 5b, c). Whereas Golden Jubilee formed basically no papillae, the other three varieties respectively responded to attempted fungal penetration at rates between 7 % and 21 %. Roughly half of these structures were effective, i.e. they blocked further fungal ingress (Fig. 5b, Additional file 3). Hypersensitive responses (HR) were only very rarely observed (<1 %) without distinct differences between the varieties. The frequencies of successful fungal penetration were significantly higher on Golden Jubilee than on B73 and Mikado. Interestingly, Farmtop allowed for higher penetration rates not only when compared to B73 but trendwise also when compared to Mikado, though the symptoms developing on Farmtop were milder than on Mikado (Fig. 5a, b). In cases of successful penetration, thick biotrophic hyphae spread beyond the first invaded epidermal cell in all varieties. Treatment of infected tissue samples with diaminobenzidine (DAB) to detect hydrogen peroxide stained host cell walls especially when conidia were found at higher density (Fig. 5d). Apparent differences between the varieties were not observed. Spray inoculation of whole plants resulted in differences in symptom severity between the four varieties that corresponded to those observed in the leaf segment assays (data not shown).

### Comparison of fungal development on different host varieties by qPCR and GFP tracing

To determine whether and how the observed differences in symptom and infection structure development on the four maize varieties corresponded to fungal biomass accumulation, we employed qPCR on DNA extracted from small excised leaf discs carrying the infection sites. Paralleling the symptom development (Fig. 5a), until 48 hpi fungal DNA remained at low, constant levels in all varieties tested (Fig. 5e). At 72 hpi, fungal DNA amounts had increased significantly in the highly susceptible variety Golden Jubilee, but also somewhat in Mikado and Farmtop. On variety B73, the onset of fungal proliferation was delayed since a substantial increase of *C. graminicola* DNA was not seen until 96 hpi. At 72 hpi and 96 hpi, fungal biomass was significantly higher in Golden Jubilee than in all other varieties. This correlates well with the early and strong symptom development and the scarceness of visible defence responses observed in this variety. However, for the variety Farmtop, both qPCR (Fig. 5e) and penetration rates (Fig. 5b) indicated a rather efficient establishment of the infection, although symptom development remained considerably weaker throughout the experiment in comparison to Golden Jubilee and Mikado. Moreover, levels of fungal DNA in variety B73 at 120 hpi (Fig. 5e) indicated growth of *C. graminicola* during later phases of infection that did, however, only led to very weak symptoms (Fig. 5a).

**Fig. 6:**
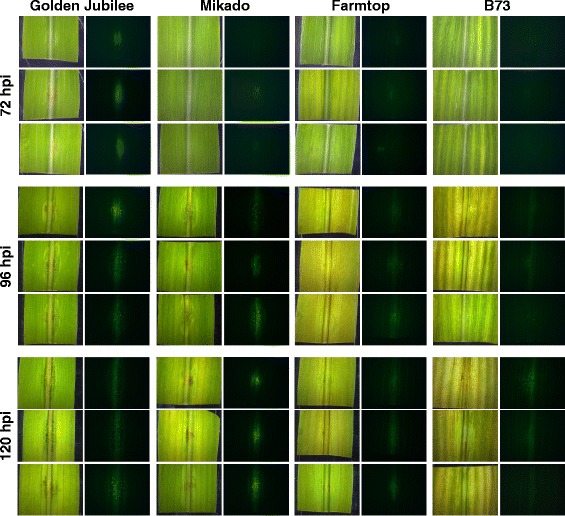
Growth of GFP-labelled *C. graminicola* on different host genotypes. Comparison of pathogenesis on four maize varieties infected by a *C. graminicola* strain constitutively expressing GFP. Infection sites were observed using a stereoscope with white light and fluorescence illumination at the indicated time points. From two experiments made, each using eight leaves per time point, three representative examples are shown per variant

To substantiate the obtained qPCR data on fungal proliferation, plant infection assays were subsequently performed with a GFP-expressing strain of *C. graminicola*. To allow for constitutive GFP expression, we created this strain by homologous integration of a cassette carrying a fusion of the eGFP marker gene to a promotor fragment of the *ToxB* gene of *Pyrenophora tritici-repentis* [13] at an intergenic locus within a genomic region that most likely comprises constitutively expressed genes [4] (Additional file 4). Virulence on corn and vegetative growth *in vitro* of this strain did not differ significantly from those of the wild type reference (not shown). Fluorescence microscopy was performed on infection sites at 72 hpi, 96 hpi and 120 hpi (Fig. 6). Matching the qPCR results (Fig. 5e), at 72 hpi clear green fluorescence signals provided evidence for a well-established and spreading *C. graminicola* infection only in Golden Jubilee. Signal strength and expansion had increased in this variety at 96 hpi. As the fungus spread longitudinally, at 120 hpi fluorescence signals arising from the inoculation point remained at similar levels.

In contrast to Golden Jubilee, fluorescence signals of the infection sites were rather weak at 72 hpi in varieties Mikado and Farmtop, indicating slower proliferation. Nevertheless, they became clearly visible at 96 hpi and had further spread at 120 hpi. Although the expansion of mycelia appeared comparable, fluorescence intensity appeared more pronounced on Mikado compared to Farmtop. This was linked to the higher number of acervuli produced on the former, which led to more focused GFP signals. In contrast to the other three varieties tested, infection of B73 with the GFP labelled strain resulted in a later and weaker emission of green fluorescence. Only at the last time point at 120 hpi, the mycelium had spread to an extent that was comparable to Mikado or Farmtop at 96 hpi reflecting the qPCR data (see Fig. 5e). Similar to Farmtop, acervuli appeared quite scarcely in B73 until 120 hpi. Taken together, we observed that the spreading of the GFP fluorescence signals reflected better the increase of fungal biomass as determined by qPCR than visual symptom development.

## Discussion

Using a qPCR assay and a GFP-labelled strain, we assessed differences in the proliferation of *C. graminicola* on several varieties of its host maize. We found that fungal biomass accumulation *in planta* varies considerably less than implied by the remarkable differences in symptom development observed on the tested maize varieties. This suggests a contribution of the host genotype to the extent of disease symptoms in this pathosystem.

Of 27 host varieties exhibiting a large range of susceptibilities to *C. graminicola* as indicated by disease severity, we have chosen four to compare the developments of fungal gDNA contents, infection structures and disease symptoms. At the high end of susceptibility is the variety Golden Jubilee followed by Mikado and then by Farmtop. Finally, we included the variety B73 since it appeared at the low end of susceptibility and since it had been used to establish the genome sequence of maize [5]. By employing qPCR and GFP experiments, we found that massive fungal proliferation indicative for necrotrophy initiated not earlier than 48 hpi on any of the four maize varieties tested. After that time, clear differences between them became obvious. Whereas the fungus did not proliferate on maize B73 until 72 hpi, it had already expanded noticeably by then on Golden Jubilee. This discrepancy correlates with the frequencies observed for successful fungal penetration and the lack of host defence reactions in the latter. One may assume that such differences of fungal proliferation *in planta* will inevitably result in corresponding differences in symptom development. However, this was not the case in the maize varieties Mikado and Farmtop that exhibited comparable fungal gDNA contents and mycelia, but remarkably different disease symptoms. Thus, fungal proliferation over time is not necessarily reflected by symptom development.

Past research had also noticed big differences in the speed and intensity of symptom development after inoculating *C. graminicola* on 183 maize varieties, which did not include any of those tested here [14]. Differences in susceptibility may link to varying rates at which phenolics accumulate in infected maize tissue [15]. Maize is known to produce a range of secondary metabolites acting as phytoalexins and phytoanticipins, such as benzoxazinones, zealexins and kauralexins [16–18]. This suggests that *C. graminicola* may encounter toxic secondary metabolites when colonising maize and it must therefore employ currently unknown mechanisms, such as degradation or extrusion to overcome their impact. Future experiments need to assess, whether the timing and the levels of secondary metabolites differ in maize varieties in such a way that it may explain the divergences observed in the proliferation of *C. graminicola in planta*.

Beyond the production of antifungal compounds, maize may attempt to ward off the invading pathogen by forming papillae or by an HR, the extent of which may vary with the host genotype. Papillae are considered a defence response of the PTI [19], which is induced when PAMPs of the invading pathogen are recognised by pattern recognition receptors (PRRs) [20, 21]. Papillae may form at some frequency even in compatible interactions. On the other hand, HR typically results from recognition of the pathogen as part of the effector-triggered immunity (ETI) [21]. Recently, it was suggested that the differences between PTI and ETI may be less distinct than initially considered [22], as some PAMPs may also provoke HR. In contrast to many other pathosystems, no clear HR has been reported for the *C. graminicola*-maize interaction, although maize is capable of HR to promote race-specific resistance to the rust *Puccinia sorghi* [23, 24].

It is not resolved whether *C. graminicola* may overcome or avoid the PTI by suppression of host defence signalling, by the masking or modification of its PAMPs or by a combination of this [9]. It is noteworthy that the high susceptibility of Golden Jubilee correlates with the lack of visible defence reactions, which may suggest that this maize variety may have a reduced ability to detect the pathogen or to mount defences that would delay fungal development in infected host tissues. In contrast to evidence of PTI occurring in the pathosystem *C. graminicola* - maize, ETI as indicated by a gene-for-gene resistance has not been reported. Using the 27 maize varieties tested in this study, we neither observed HR as a visible sign of gene-for-gene resistance.

Support for the notion of an active plant defence even during the biotrophic phase of *C. graminicola* came from work showing that the transcript levels of some defence-related genes such as *ZmPR1*, *ZmPR4b*, *ZmPR5* and *ZmAChit* begin to rise as early as 24 hpi [7]. Moreover, a study on 44 defence- and stress-related genes in infected maize leaves confirmed transcript levels of *ZmPR1*, *ZmPR3*, *ZmPR5* and *ZmPR10* to increase already during the biotrophic phase of *C. graminicola*, whereas others, such as *ZmBx1*, *ZmIGL* and *ZmCHS C2*, were found decreased [25]. The latter encode enzymes involved in the synthesis of phytoalexins and phytoanticipins. Metabolome profiling of infected leaves performed in the same study suggested that maize is able to mount defence reactions already during the biotrophic phase but *C. graminicola* seems to be able to avoid or overcome them eventually.

*Ustilago maydis*, which also infects maize, has a much longer biotrophic phase than *C. graminicola*. Nevertheless, during the earliest phase of infection this basidiomycete also evokes responses in the host such as autofluorescence and transcriptional induction of defence-related genes indicating the recognition of the pathogen [26]. However, many of these genes were downregulated after 24 h suggesting that *U. maydis* had initiated the suppression of the host´s PTI. Further evidence for suppression by *U. maydis* came from secreted effectors targeting distinct defence pathways of maize [27–29]. Another study found suppression of *PR-1* and *TPS6* (sesquiterpene cyclase) in maize leaves infected with *U. maydis* but not with *C. graminicola* [30]. In consideration of these studies, defence suppression may play a lesser role in *C. graminicola* than in *U. maydis*, which infects the same host. However, since reports from other *Colletotrichum* species indicated a contribution of defence suppression in their interactions with compatible hosts it remains unsettled to which degree this mechanism is important for the pathogenesis of *C. graminicola* [31–33].

Another possible way to establish a compatible interaction despite the host´s PTI is its avoidance through the modification of PAMPs such as fungal chitin, β-1,3-glucan and ergosterol [9, 22, 34–36]. For instance, LysM effectors that were also found in *Colletotrichum* spp. bind chitin fragments to prevent the elicitation of PTI [37]. Additional evidence in *C. graminicola* for an avoidance of PTI is the conversion of chitin to chitosan after penetration of the host surface [38] and the down-regulation of β-glucan synthesis during biotrophy [9]. Thus, avoidance contributes to the biotrophy of *C. graminicola* by diminishing the PTI of maize.

In this study, we designed and validated a qPCR assay that offers high sensitivity and specificity for the measurement of fungal biomass in infected plant tissues. Although fungal-specific molecules such as ergosterol, chitin and certain phospholipid fatty acids have been used to estimate biomass, this approach requires that the amounts of these biomarkers do not vary between different fungal cell types formed during pathogenesis and between different physiological conditions, assumptions that must be treated with caution [11, 39, 40]. Several reports compared the reliability of qPCR-based biomass determination to those using the mentioned biomarkers. Most of these concluded that qPCR is superior, others found both approaches appropriate [39–46].

## Conclusions

This study shows that development of *C. graminicola* in infected maize tissues can be better estimated from qPCR and GFP tracing experiments than from macroscopic symptom development. The new qPCR assay will be useful in several circumstances. First, it can reveal relatively subtle differences between genetically engineered mutants and the wild type reference with respect to the development of fungal biomass during pathogenesis. This will be valuable for the analysis of candidate genes, which may only have a minor contribution to overall virulence. Second, it may be important to assess specifically the effect of an introduced mutation on several host varieties in order to identify reliably all possible deviations from the wild type reference. Finally, also plant breeders screening in maize for an improved resistance against *C. graminicola* may consider that visual inspection of symptom development may not suffice to select optimal host genotypes.

## Methods

### Plant and fungal cultivation

Fungal strains included the wild-type reference strain CgM1.001 (formerly CgM2) of *Colletotrichum graminicola* (Ces.) Wilson (teleomorph *Glomerella graminicola* (Politis)) and mutant strains derived thereof by *Agrobacterium tumefaciens*-mediated transformation (ATMT) [12]. Mutant strains, which were originally assigned as AT2.463, AT2.374 and AT2.276, were designated as AT-1, AT-2 and AT-3 throughout this study.

Varieties of maize (*Zea mays* L.) used for inoculation were B73 (ARS-USDA, Ames, Iowa, USA), Mikado (KWS Saat AG, Einbeck, Germany), Golden Jubilee (Territorial Seed Company, Cottage Grove, OR) and Farmtop (FarmSaat AG, Everswinkel, Germany). Further varieties used in Fig. 4 are listed in Additional file 2.

Conditions for cultivation of the fungus and maize in the greenhouse as well as in environmentally controlled growth chambers had been described [8].

### Creation of a *C. graminicola* GFP strain

For construction of a *C. graminicola* strain carrying an eGFP marker cassette whose expression levels are only minimally influenced by the genomic position of the integration site and developmental stage the annotated genome [4] was screened for loci fulfilling as fully as possible the following conditions. First, the site chosen for the targeted integration of the cassette should be an intergenic region between 1 and 2 kb in length to allow for sufficiently long flanking regions for homologous recombination that do not overlap with coding sequences while minimising the risk of presence of unidentified features that could lead to phenotypes when disrupted. For this second reason, the genes on the left and on the right should also preferably have a convergent orientation. Finally, the integration site should reside in a genomic region rich in more or less constitutively expressed genes indicating a constitutively euchromatic state, which was examined on the basis of gene annotations and published transcriptome data [4]. One such locus, between genes GLRG_03632 and GLRG_03633 on Supercontig 11 (Additional file 4) was chosen as target region. The eGFP expression cassette was constructed using the Golden Gate system for one-step restriction/ligation cloning of multiple fragments [47]. Left and right flanks of the target site were amplified from CgM1.001 genomic DNA using primer pairs BSA-Ekt5'-pBR322 and BSA-Ekt5'-Prom and, respectively, BSA-Ekt3'-G418 and BSA-Ekt3'-pBR322 (Additional file 5). The eGFP encoding ORF was amplified from plasmid pSM1 [48] using primers BSA-GFP.F1-Prom and BSA-GFP.R3-Tnos. The strong constitutive ToxB promoter from *Pyrenophora tritici-repentis*, chosen to drive eGFP expression, was amplified from 1026244_P-ToxB_pMA-T (Susanne Köllmer, Martin-Luther-Universität Halle, unpublished) using primers BSA-PtoxA-Ekt5' and BSA-ToxA.R1-GFP. The *nos* terminator was amplified from p123-mcherry [49] using primers BSA-Tnos.F1-GFP and BSA-Tnos.R2-Sel. A geneticin (G418) resistance cassette, consisting of the neomycin phosphotransferase II (*nptII*) gene under control of the *Aspergillus nidulans trpC* promoter and terminator, was amplified from plasmid pII99 [50] using primers BSA-G418.F1-Tnos and BSA-G418-Ekt3'. The target vector for insertion of multi-fragment constructs by Golden Gate cloning, pBR322-Bsa, was generated by amplifying pBR322 with primers pBR322-For and pBR322-Rev, followed by *Xho*I-digestion of the PCR product and self-ligation. Finally, pBR322-Bsa and the PtoxB, eGFP, Tnos and resistance cassette fragments were mixed in equimolar concentrations and *Bsa*I-digested/ligated [47]. After transformation in chemocompetent *E. coli* DH5alpha, plasmids were purified and verified by restriction digestion and PCR analyses. Correct plasmids were pooled and used as PCR template with primers Uni-Ekt-For and Uni-Ekt-Rev to generate a linear fragment that was transformed into *C. graminicola* M1.001 employing established procedures [51]. All primers were synthesised by biomers.net GmbH (Ulm, Germany) and are listed in Additional file 5.

### Plant infection assay

Infection of maize by *C. graminicola* strains was assessed using a detached leaf assay. Fourteen days after sowing, segments (~8 cm) of third leaves were harvested and placed onto moistened filter paper in plastic petri dishes of 14 cm diameter. Fungal inoculum was produced by washing conidia off from OMA plates with 0.02 % (v/v) Tween 20. For qPCR analysis, 10 μl droplets of a conidial suspension adjusted to 10^6^ conidia/ml were inoculated, the petri dishes were sealed with parafilm and incubated at 23 °C in the dark for up to 120 h. Infected tissue samples were harvested with a cork borer having a diameter of 8 mm. Finally, pools comprising five to eight tissue discs were frozen in liquid nitrogen and stored at −80 °C. For microscopic analysis (see below), inoculation applied essentially the same procedure but used suspensions of 10^5^ conidia/ml.

### Isolation of genomic DNA

Frozen fungal or plant material was ground in a TissueLyser (QIAGEN GmbH AG, Hilden, Germany) for 30 s at 30 Hz by two steel balls (HECHT Kugellager GmbH & Co. KG, Winnenden, Germany) having diameters of 3 mm. Isolation of total genomic DNA utilised the peqGOLD Plant DNA Mini Kit (PEQLAB Biotechnologie GmbH, Erlangen, Germany). Just before the addition of the first buffer of the kit, the ground samples were spiked with 10 μl of pUC18 (Thermo Fisher Scientific) adjusted to 5 pg/μl. This served as an external standard to control and to correct for putative DNA losses throughout the extraction procedure. Correction was achieved by assessing and applying sample-specific qPCR normalization factors as given below. The final volume of each DNA preparation was 100 μl. Concentration of the DNA preparation was assessed by a NanoDrop 1000 spectrophotometer (Thermo Fisher Scientific Inc., Waltham, MA, USA).

### Design and validation of primers for qPCR

Due to its multi-copy occurrence in the genome promoting an increased sensitivity of the assay and its specificity, the internal transcribed spacer (ITS) of the ribosomal RNA-coding DNA (= rDNA) was chosen as the target for qPCR amplification. Based on the rDNA sequences of *C. graminicola* (GenBank: EU400146.1) and *Z. mays* (GenBank: DQ683016.1), several ITS1 and ITS2 primer pairs specific to *C. graminicola* were derived using the software Clone Manager (version 9.0; Scientific & Educational Software, Cary, NC, USA). Primers were designed to amplify fragments of about 100 bp and to have annealing temperatures of 60 °C, which is the recommended temperature for the used qPCR kit (see below). In conventional PCR pre-tests (35 cycles) that used DreamTaq DNA polymerase (Thermo Fisher Scientific), the primer pairs were analysed for putative cross-reactions with the non-template DNAs from *Z. mays* (uninfected) and pUC18. The primer pair producing optimal results was Cg_ITS2-F1.1 and Cg_ITS2-R1 and was thus further on applied. The primers M13new-For and M13new-Rev were confirmed to be specific for pUC18 excluding the possibility of cross-reactions with the gDNA of *C. graminicola* and *Z. mays*. All primers were synthesised by biomers.net GmbH (Ulm, Germany) and are listed in Additional file 5.

### qPCR analysis

We employed the Power SYBR® Green PCR Master Mix (Applied Biosystems GmbH, Darmstadt, Germany) and the iCycler model MyiQ Single color (Bio-Rad Laboratories GmbH, München, Germany) according to manufacturer's protocols. C_T_ values that were the basis for subsequent quantifications were assessed by the iCycler software iQ5 (version 2.0, Bio-Rad). Reaction volumes of 20 μl included 1 μl of diluted DNA template and 0.1 μM of each primer. Reaction conditions were 10 min at 95 °C for the activation of the polymerase, followed by 50 cycles of 95 °C for 15 s and 60 °C for 60 s with data collection.

To establish calibration curves, serial dilutions of either pure fungal gDNA ranging from 2 pg/μl to 20 ng/μl, or pure pUC18 plasmid DNA (Thermo Fisher Scientific) ranging from 0.5 fg/μl to 5 ng/μl were mixed with a constant amount of 10 ng of genomic DNA isolated from uninfected maize leaves. Calibration curves were assessed by calculating the linear function relating C_T_ values (averaged from 3 technical repeats) to their corresponding logarithmic transformed DNA amounts. In addition to the C_T_ values, the RFU (= Relative Fluorescence units) threshold values for each of the two PCR experiments have been assessed by iQ5 analysis, i.e. 59.3 (*C. graminicola* gDNA), respectively 143.35 (pUC18). Furthermore, reaction efficiencies were calculated with the formula E = (10^−1/m^-1)x100%, where m described the slope of the calibration curve.

For the absolute quantification of fungal DNA in infected tissue samples, for each DNA preparation two qPCR reactions were run separately, i.e. one applying the primer pair Cg_ITS2-F1.1 and Cg_ITS2-R1, and the second M13new-For and M13new-Rev. Prior to qPCR analysis, genomic DNA preparations were diluted to 10–20 ng/μl. Raw data were adjusted by applying the same threshold values applied to calculate the respective calibration curves (see above). The resulting C_T_ values were then used to calculate the absolute amount of target DNA in a given reaction. Based on the expectation that 1 μl of undiluted sample DNA would contain 0.5 pg of plasmid DNA if the recovery rate would be 100 %, sample specific correction factors were determined as the ratio of the expected to the obtained pUC18 concentration. Finally, the correction as well as the dilution factors were applied to calculate the total amount of *C. graminicola* DNA in each leaf sample comprising six leaf discs as mentioned above. Calculations and subsequent statistical analyses employed the softwares Microsoft Excel 2007 (Microsoft, Redmond, USA) and XLSTAT (Addinsoft, Andernach, Germany).

### Microscopy

Fungal morphogenesis in infected maize was examined microscopically using tissues samples from detached leaf assays as described above. Prior to observation, samples were fixed and bleached in ethanol-acetic acid (3:1, v/v) for 24 h and then stained in 0.1 % aniline blue for 10 min. After washing for 5 min in water, samples were stored in 20 % glycerol at 4 °C. Bright field and fluorescence microscopy employed a Nikon Eclipse 600 microscope equipped with appropriate filter systems (Nikon GmbH, Düsseldorf, Germany). Photographs were taken with a CCD-camera Digital Sight DS-Fi1 (Nikon) and were analysed with the software NIS-Elements D (version 2.30, Nikon). Production of hydrogen peroxide in infected tissues was visualised by DAB staining as reported previously [52].

## Additional files

The following Additional files are available with this paper. Additional file 1 documents the specificity of the primers used for qPCR. Additional file 2 is a table listing the maize varieties tested for susceptibility against *C. graminicola*. Additional file 3 shows micrographs documenting effective and ineffective papillae formed by maize in response to *C. graminicola*. Additional file 4 is a graphical representation of the genomic insertion site for an eGFP expression cassette and of the structure of the expression cassette. Additional file 5 is a table listing all PCR-primers used in this work. Additional file 1: Specificity of qPCR. Primers employed were (A) Cg_ITS2-F1.1/Cg_ITS2-R1 and (B) M13new-For/M13new-Rev. Conventional PCR (35 cycles) using as template 10 ng of gDNA of *C. graminicola* (lane 1) and *Zea mays* (lane 2), 50 pg of pUC18 (lane3), 10 ng of each gDNA of *C. graminicola* and *Z. mays* (lane 4), 10 ng of each gDNA of *C. graminicola* and *Z. mays* plus 50 pg of pUC18 (lane 5), and water as a non-template control (lane 6). A low molecular weight ladder was included in lane 7 on a 1.5 % (w/v) Sodium borate-Agarose gel. (PPTX 314 kb) Additional file 2: Maize varieties. Maize varieties tested for susceptibility against *C. graminicola* by supplier. (DOCX 17 kb) Additional file 3: Differentiation of effective and ineffective papillae formed by maize in response to attempted penetration by the *C. graminicola* wild type reference. Bright field microscopy, serial focussing allowed to follow fungal morphogenesis. Magnification is 600x. Bars represent 50 μm. (PPTX 28444 kb) Additional file 4: Construction of a *C. graminicola* strain constitutively expressing eGFP. Graphical representation of the genomic region from base pair 275,656 to base pair 295830, containing annotated genes GLRG_03629 to GLRG_03636 on supercontig 11 where the eGFP expression cassette was inserted. Annotations are those provided by the Broad Institute (Broad) or based on NCBI protein Blast best hits (BlastP) for genes annotated as hypothetical by the Broad Institute. Transcript abundances in appressoria (AP), during the biotrophic phase of fungal development (BP) and during the necrotrophic phase (NP) were taken from [4]. The insert combines an eGFP expression unit consisting of the *P. tritici-repentis ToxB* promoter (PtoxB), the eGFP coding sequence and the *Agrobacterium tumefaciens nos* terminator (Tnos) with a geneticin/G418/neomycin resistance unit consisting of the promoter and terminator of *Aspergillus nidulans trpC* flanking the transposon Tn5 *npt*II gene. Flanking sequences homologous to the intergenic region between GLRG_03632 and GLRG_03633 allow targeted integration of the construct. (PPTX 77 kb) Additional file 5: Primers used. Primers used for the construction of the eGFP expression cassette and the qPCR assay for fungal biomass. Uppercase: target sequence-specific. Bold: *Bsa*I recognition sequence. Underlined: *Bsa*I cleavage site, yielding a 5'-overhang. Italics: *Xho*I cleavage site for generation of pBR322-Bsa from pBR322. *: Two site-directed mutagenic bases (lowercase) in the target-specific sequence for removal of plasmid-borne *Bsa*I recognition sequence. (DOCX 16 kb)
