# Supplementary figures and images for: Correspondence between symptom development of Colletotrichum graminicola and fungal biomass, quantified by a newly developed qPCR assay, depends on the maize variety
Source: BMC Microbiol. 2016 May 23;16:94. doi: 10.1186/s12866-016-0709-4 (PMC4877754; doi:10.1186/s12866-016-0709-4)

## Slide 1
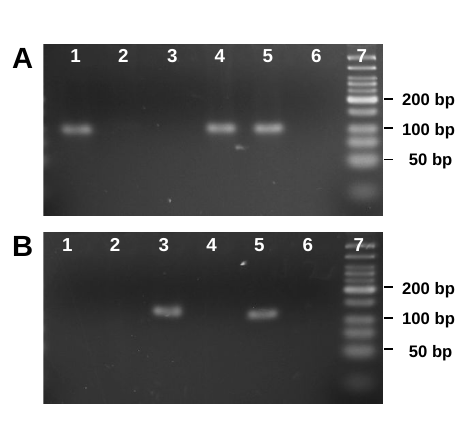

A
1
2
3
4
5
6
7
200 bp
100 bp
 50 bp
B
1
2
3
4
5
6
7
200 bp
100 bp
 50 bp

Supplement: Additional file 1: — Specificity of qPCR. Primers employed were (A) Cg_ITS2-F1.1/Cg_ITS2-R1 and (B) M13new-For/M13new-Rev. Conventional PCR (35 cycles) using as template 10 ng of gDNA of C. graminicola (lane 1) and Zea mays (lane 2), 50 pg of pUC18 (lane3), 10 ng of each gDNA of C. graminicola and Z. mays (lane 4), 10 ng of each gDNA of C. graminicola and Z. mays plus 50 pg of pUC18 (lane 5), and water as a non-template control (lane 6). A low molecular weight ladder was included in lane 7 on a 1.5 % (w/v) Sodium borate-Agarose gel. (PPTX 314 kb) [file 12866_2016_709_MOESM1_ESM.pptx]
