# Supplementary material for: Correspondence between symptom development of Colletotrichum graminicola and fungal biomass, quantified by a newly developed qPCR assay, depends on the maize variety
Source: BMC Microbiol. 2016 May 23;16:94. doi: 10.1186/s12866-016-0709-4 (PMC4877754; doi:10.1186/s12866-016-0709-4)
Supplement: Additional file 2: — Maize varieties. Maize varieties tested for susceptibility against C. graminicola by supplier. (DOCX 17 kb) [file 12866_2016_709_MOESM2_ESM.docx]

**Additional file 2** Maize varieties tested for susceptibility

| **Name** | **Type** | **Supplier** |
| --- | --- | --- |
| Ajaxx | SC | RAGT Saaten Deutschland GmbH  (Herford, Germany) |
| Alumic | SC |  |
| Coxximo | SC |  |
| Könixx | TWC |  |
| Saludo | TWC |  |
| Shexxpir | SC |  |
| Sphinxx | SC |  |
| Taxxi | SC |  |
| Tiberio | TWC |  |
| Xxira | SC |  |
| PR39K13 | SC | Pioneer Hi-Bred GmbH  (Buxtehude, Germany) |
| PR39F56 | SC/BT |  |
| PR39F58 | SC |  |
| PR39V17 | SC/BT |  |
| DKC2864 | SC | Monsanto Agrar Deutschland GmbH  (Düsseldorf, Germany) |
| DKC4371 | SC |  |
| DK315 | SC |  |
| NK Nekta | SC | Syngenta Seeds GmbH  (Bad Salzuflen, Germany) |
| NK Winn | SC |  |
| Oldham | SC |  |
| Farmoso | SC | FarmSaat AG  (Everswinkel, Germany) |
| Farmtop | SC |  |
| Mikado | n.d. | KWS Saat AG  (Einbeck, Germany) |
| Nathan | SC |  |
| ES Paroli | SC | EURALIS Saaten GmbH  (Norderstedt, Germany) |
| Golden Jubilee | SC | West Coast Seeds Inc.  (Delta, Canada) |
| B73 | I | ARS-USDA  (Ames, Iowa, USA) |

SC = Single-Cross hybrid

TWC = Three-way-cross hybrid

BT = BT-toxin containing transgene maize

I = Inbred
