# Supplementary material for: Correspondence between symptom development of Colletotrichum graminicola and fungal biomass, quantified by a newly developed qPCR assay, depends on the maize variety
Source: BMC Microbiol. 2016 May 23;16:94. doi: 10.1186/s12866-016-0709-4 (PMC4877754; doi:10.1186/s12866-016-0709-4)
Supplement: Additional file 3: — Differentiation of effective and ineffective papillae formed by maize in response to attempted penetration by the C. graminicola wild type reference. Bright field microscopy, serial focussing allowed to follow fungal morphogenesis. Magnification is 600x. Bars represent 50 μm. (PPTX 28444 kb) [file 12866_2016_709_MOESM3_ESM.pptx]

## Slide 1
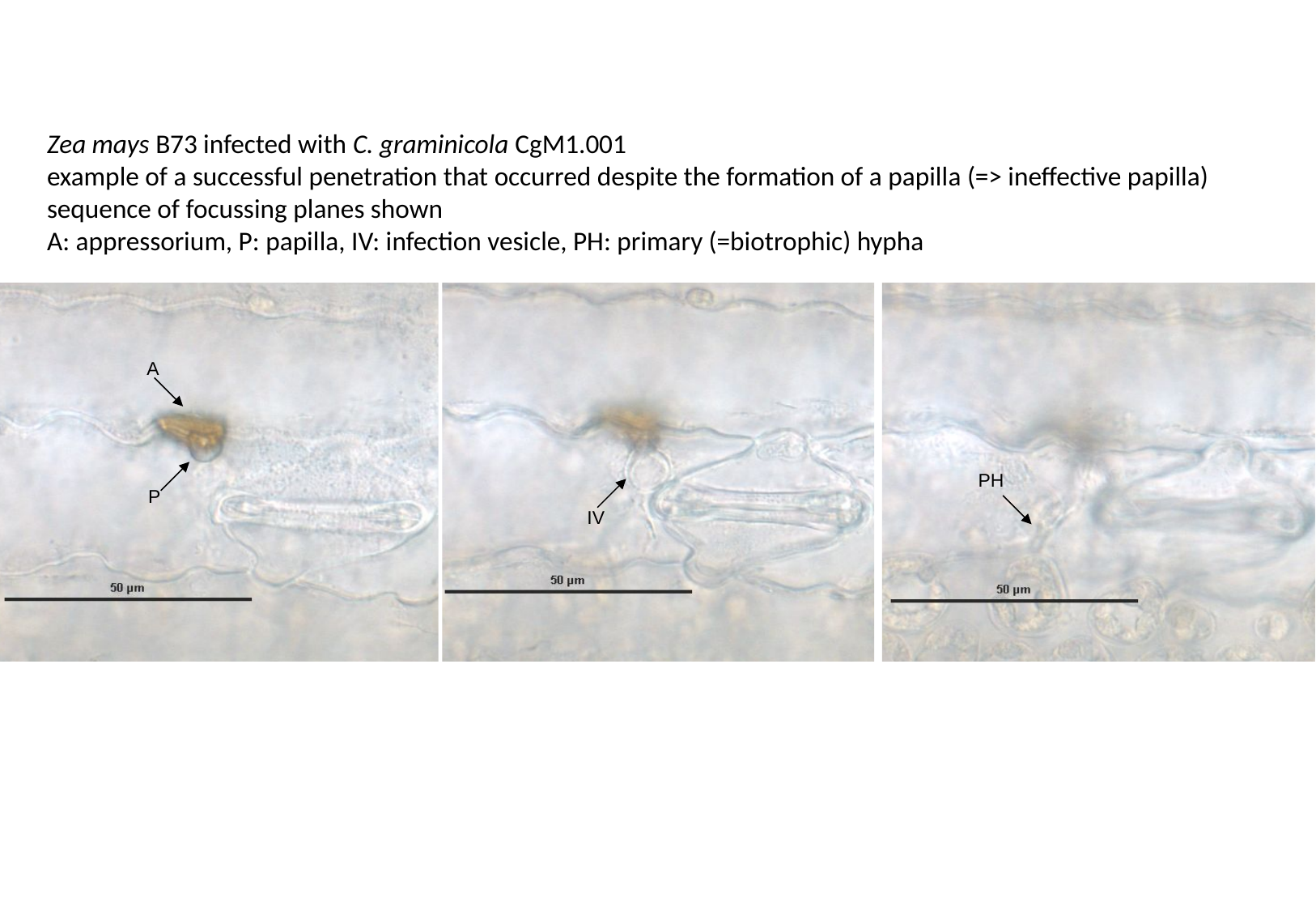

Zea mays B73 infected with C. graminicola CgM1.001
example of a successful penetration that occurred despite the formation of a papilla (=> ineffective papilla)
sequence of focussing planes shown
A: appressorium, P: papilla, IV: infection vesicle, PH: primary (=biotrophic) hypha
A
PH
P
IV

## Slide 2
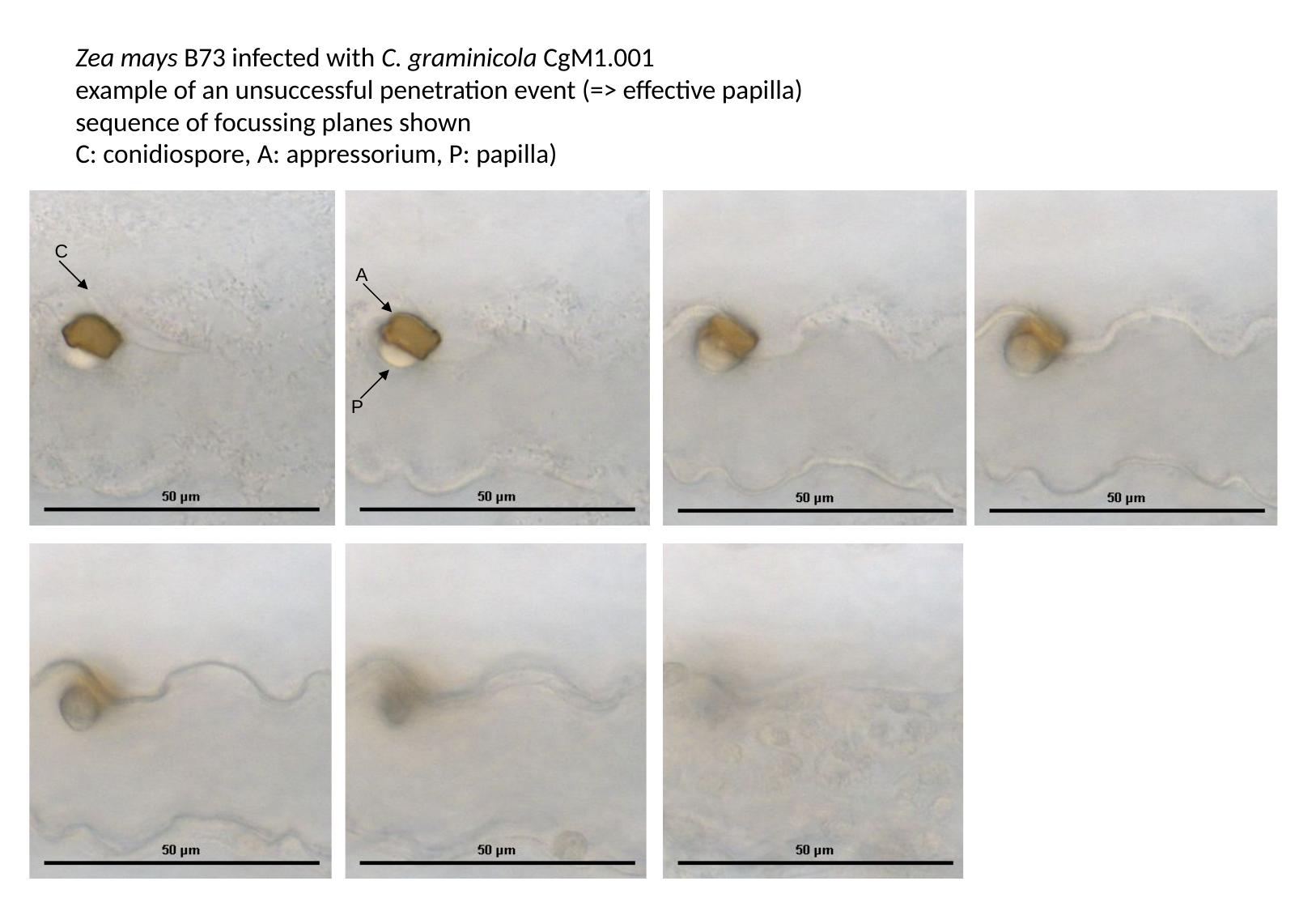

Zea mays B73 infected with C. graminicola CgM1.001
example of an unsuccessful penetration event (=> effective papilla)
sequence of focussing planes shown
C: conidiospore, A: appressorium, P: papilla)
C
A
P
