# Supplementary material for: Correspondence between symptom development of Colletotrichum graminicola and fungal biomass, quantified by a newly developed qPCR assay, depends on the maize variety
Source: BMC Microbiol. 2016 May 23;16:94. doi: 10.1186/s12866-016-0709-4 (PMC4877754; doi:10.1186/s12866-016-0709-4)
Supplement: Additional file 4: — Construction of a C. graminicola strain constitutively expressing eGFP. Graphical representation of the genomic region from base pair 275,656 to base pair 295830, containing annotated genes GLRG_03629 to GLRG_03636 on supercontig 11 where the eGFP expression cassette was inserted. Annotations are those provided by the Broad Institute (Broad) or based on NCBI protein Blast best hits (BlastP) for genes annotated as hypothetical by the Broad Institute. Transcript abundances in appressoria (AP), during the biotrophic phase of fungal development (BP) and during the necrotrophic phase (NP) were taken from [4]. The insert combines an eGFP expression unit consisting of the P. tritici-repentis ToxB promoter (PtoxB), the eGFP coding sequence and the Agrobacterium tumefaciens nos terminator (Tnos) with a geneticin/G418/neomycin resistance unit consisting of the promoter and terminator of Aspergillus nidulans trpC flanking the transposon Tn5 nptII gene. Flanking sequences homologous to the intergenic region between GLRG_03632 and GLRG_03633 allow targeted integration of the construct. (PPTX 77 kb) [file 12866_2016_709_MOESM4_ESM.pptx]

## Slide 1
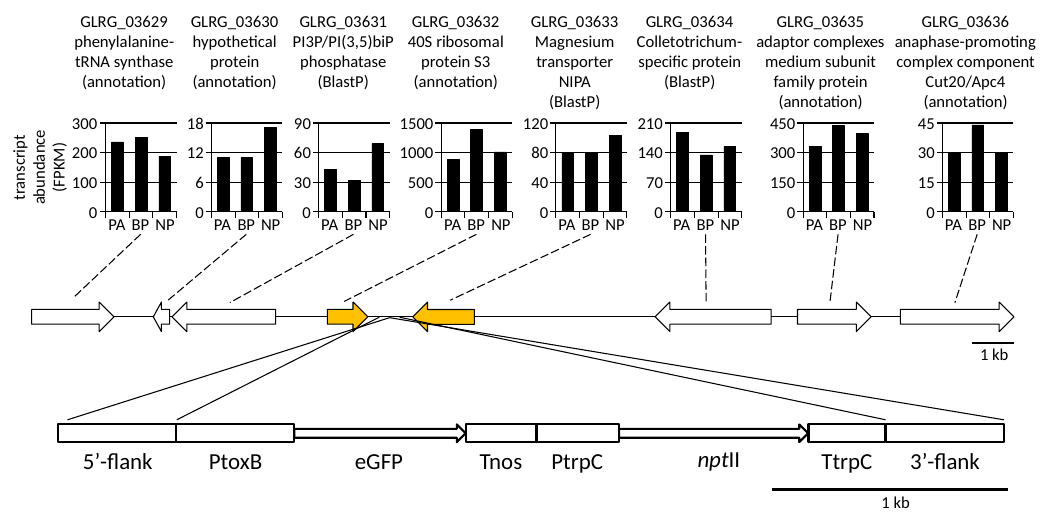

GLRG_03629
phenylalanine-
tRNA synthase (annotation)
GLRG_03630
hypothetical
protein
(annotation)
GLRG_03631
PI3P/PI(3,5)biP
phosphatase
(BlastP)
GLRG_03632
40S ribosomal
protein S3
(annotation)
GLRG_03633
Magnesium
transporter
NIPA
(BlastP)
GLRG_03634
Colletotrichum-
specific protein
(BlastP)
GLRG_03635
adaptor complexes
medium subunit
family protein
(annotation)
GLRG_03636
anaphase-promoting
complex component
Cut20/Apc4
(annotation)
300
200
100
0
PA
BP
NP
18
12
6
0
PA
BP
NP
90
60
30
0
PA
BP
NP
1500
1000
500
0
PA
BP
NP
120
80
40
0
PA
BP
NP
210
140
70
0
PA
BP
NP
450
300
150
0
PA
BP
NP
45
30
15
0
PA
BP
NP
transcript
abundance
(FPKM)
1 kb
nptII
5’-flank
PtoxB
eGFP
Tnos
PtrpC
TtrpC
3’-flank
1 kb
