# Supplementary material for: Correspondence between symptom development of Colletotrichum graminicola and fungal biomass, quantified by a newly developed qPCR assay, depends on the maize variety
Source: BMC Microbiol. 2016 May 23;16:94. doi: 10.1186/s12866-016-0709-4 (PMC4877754; doi:10.1186/s12866-016-0709-4)
Supplement: Additional file 5: — Primers used. Primers used for the construction of the eGFP expression cassette and the qPCR assay for fungal biomass. Uppercase: target sequence-specific. Bold: BsaI recognition sequence. Underlined: BsaI cleavage site, yielding a 5'-overhang. Italics: XhoI cleavage site for generation of pBR322-Bsa from pBR322. *: Two site-directed mutagenic bases (lowercase) in the target-specific sequence for removal of plasmid-borne BsaI recognition sequence. (DOCX 16 kb) [file 12866_2016_709_MOESM5_ESM.docx]

**Additional file 5** Primers used

| **Primer name** | **5'-primer sequence-3'** | **Target molecule** |
| --- | --- | --- |
| BSA-Ekt5'-pBR322 | ttt**ggtctc**aaggTGTCGTCGAGGAGACTGCTGAG | *C. graminicola* M1.001 gDNA |
| BSA-Ekt5'-prom | aaa**ggtctc**agtgtCAGGAGCCTGGCGAATTGAG | *C. graminicola* M1.001 gDNA |
| BSA-Ekt3'-G418 | ttt**ggtctc**aggagGCTCCGGTCTTACGTCTATG | *C. graminicola* M1.001 gDNA |
| BSA-Ekt3'-pBR322 | aa**ggtctc**aaagcGTACAGTGGAACCGTCTACTC | *C. graminicola* M1.001 gDNA |
| BSA-GFP.F1-Prom | ttt**ggtctc**aaccgATGGTGAGCAAGGGCGAGGAG | pSM1 |
| BSA-GFP.R3-Tnos | aaa**ggtctc**agccgTTACTTGTACAGCTCGTCCATGCC | pSM1 |
| BSA-Ptox-Ekt5' | ttt**ggtctc**aacacAGGCCACGTGTCTTGTCCAG | 1026222_P-ToxB_pMA-T |
| BSA-ToxA.R1-GFP | aaa**ggtctc**acggtAGGCCAGTCTTGTGCTCCAG | 1026222_P-ToxB_pMA-T |
| BSA-Tnos.F1-GFP | ttt**ggtctc**acggcGGCATGGACGAGCTGTACAAG | p123-mcherry |
| BSA-Tnos.R2-Sel | aaa**ggtctc**actgaCGACTCACTATAGGaAGgCCG* | p123-mcherry |
| BSA-G418.F1-Tnos | ttt**ggtctc**atcagCTTGGCTGGAGCTAGTGGAGGT | pII99 |
| BSA-G418-Ekt3' | aaa**ggtctc**actccCCGTTGATCTGCTTGATCTC | pII99 |
| Uni-Ekt-For | TCGTCGAGGAGACTGCTGAG | targeted integrative cassettes |
| Uni-Ekt-Rev | GTACAGTGGAACCGTCTACTC | targeted integrative cassettes |
| pBR322-For | ctga*ctcgag*tcagttgcac**ggtctc**agctTCACGAGGCCCTTTCGTC | pBR322 |
| pBR322-Rev | gcta*ctcgag*cagtcgtaca**ggtctc**aacctGTGCCTCACTGATTAAGCATTG | pBR322 |
| Cg_ITS2-F1.1 | CGTCGTAGGCCCTTAAAGGTAG | *C. graminicola* M1.001 ITS2 |
| Cg_ITS2-R1 | TTACGGCAAGAGTCCCTC | *C. graminicola* M1.001 ITS2 |
| M13new-For | GTAAAACGACGGCCAGTGC | pUC18 |
| M13new-Rev | CACAGGAAACAGCTATGACC | pUC18 |

Uppercase: target-specific sequence

Bold: *Bsa*I recognition sequence

Underlined: sequence digested to 5'-overhang by *Bsa*I

Italics: *Xho*I cleavage sites used for generation of pBR322-Bsa

* Two site-directed mutagenic bases (lowercase) in the target-specific sequence for removal of plasmid-borne *Bsa*I recognition sequence
